# Supplementary material for: OpenMM 7: Rapid development of high performance algorithms for molecular dynamics
Source: PLoS Comput Biol. 2017 Jul 26;13(7):e1005659. doi: 10.1371/journal.pcbi.1005659 (PMC5549999; doi:10.1371/journal.pcbi.1005659)
Supplement: S1 Source Code — (ZIP) [file pcbi.1005659.s002.zip › OpenMM-7.0.1-Source/docs/api-c++/generated/OpenMM.BrownianIntegrator.html]

BrownianIntegrator — OpenMM @OPENMM\_MAJOR\_VERSION@.@OPENMM\_MINOR\_VERSION@ documentation


# BrownianIntegrator¶

*class* `OpenMM::``BrownianIntegrator`¶
:   This is an `Integrator` which simulates a `System` using Brownian dynamics.

    Methods

    |  |  |
    | --- | --- |
    | `BrownianIntegrator` | Create a `BrownianIntegrator`. |
    | `getTemperature` | Get the temperature of the heat bath (in Kelvin). |
    | `setTemperature` | Set the temperature of the heat bath (in Kelvin). |
    | `getFriction` | Get the friction coefficient which determines how strongly the system is coupled to the heat bath (in inverse ps). |
    | `setFriction` | Set the friction coefficient which determines how strongly the system is coupled to the heat bath (in inverse ps). |
    | `getRandomNumberSeed` | Get the random number seed. |
    | `setRandomNumberSeed` | Set the random number seed. |
    | `step` | Advance a simulation through time by taking a series of time steps. |

    `BrownianIntegrator`(double *temperature*, double *frictionCoeff*, double *stepSize*)¶
    :   Create a `BrownianIntegrator`.

        |  |  |
        | --- | --- |
        | Parameters: |  |

        - **temperature** – the temperature of the heat bath (in Kelvin)
        - **frictionCoeff** – the friction coefficient which couples the system to the heat bath, measured in 1/ps
        - **stepSize** – the step size with which to integrate the system (in picoseconds)

    double `getTemperature`() *const*¶
    :   Get the temperature of the heat bath (in Kelvin).

        |  |  |
        | --- | --- |
        | Returns: | the temperature of the heat bath (in Kelvin). |

    void `setTemperature`(double *temp*)¶
    :   Set the temperature of the heat bath (in Kelvin).

        |  |  |
        | --- | --- |
        | Parameters: |  |

        - **temp** – the temperature of the heat bath, measured in Kelvin.

    double `getFriction`() *const*¶
    :   Get the friction coefficient which determines how strongly the system is coupled to the heat bath (in inverse ps).

        |  |  |
        | --- | --- |
        | Returns: | the friction coefficient, measured in 1/ps |

    void `setFriction`(double *coeff*)¶
    :   Set the friction coefficient which determines how strongly the system is coupled to the heat bath (in inverse ps).

        |  |  |
        | --- | --- |
        | Parameters: |  |

        - **coeff** – the friction coefficient, measured in 1/ps

    int `getRandomNumberSeed`() *const*¶
    :   Get the random number seed. See `setRandomNumberSeed()` for details.

    void `setRandomNumberSeed`(int *seed*)¶
    :   Set the random number seed. The precise meaning of this parameter is undefined, and is left up to each `Platform` to interpret in an appropriate way. It is guaranteed that if two simulations are run with different random number seeds, the sequence of random forces will be different. On the other hand, no guarantees are made about the behavior of simulations that use the same seed. In particular, Platforms are permitted to use non-deterministic algorithms which produce different results on successive runs, even if those runs were initialized identically.

        If seed is set to 0 (which is the default value assigned), a unique seed is chosen when a `Context` is created from this `Force`. This is done to ensure that each `Context` receives unique random seeds without you needing to set them explicitly.

    void `step`(int *steps*)¶
    :   Advance a simulation through time by taking a series of time steps.

        |  |  |
        | --- | --- |
        | Parameters: |  |

        - **steps** – the number of time steps to take

High performance molecular simulation on GPUs


### Navigation

- Core Objects
- Forces
- Integrators
- Extras

©2015, Stanford University and the Authors.
|
Powered by Sphinx 1.3.1
& Alabaster 0.7.7
|
Page source
